# Magnitude of risks and benefits of the addition of bevacizumab to chemotherapy for advanced breast cancer patients: Meta-regression analysis of randomized trials

**DOI:** 10.1186/1756-9966-30-54

**Published:** 2011-05-12

**Authors:** Federica Cuppone, Emilio Bria, Vanja Vaccaro, Fabio Puglisi, Alessandra Fabi, Isabella Sperduti, Paolo Carlini, Michele Milella, Cecilia Nisticò, Michelangelo Russillo, Paola Papaldo, Gianluigi Ferretti, Matti Aapro, Diana Giannarelli, Francesco Cognetti

**Affiliations:** 1 Department of Medical Oncology, Regina Elena National Cancer Institute, Roma, Italy; 2 Medical Oncology, University of Verona, Italy; 3 Department of Medical Oncology, University Hospital of Udine, Udine, Italy; 4 Biostatistics/Scientific Direction, Regina Elena National Cancer Institute, Roma, Italy; 5 Institut Multidisciplinaire d'Oncologie, Clinique de Genolier, Genolier, Switzerland

## Abstract

**Background:**

Although the addition of bevacizumab significantly improves the efficacy of chemotherapy for advanced breast cancer, regulatory concerns still exist with regard to the magnitude of the benefits and the overall safety profile.

**Methods:**

A literature-based meta-analysis to quantify the magnitude of benefit and safety of adding bevacizumab to chemotherapy for advanced breast cancer patients was conducted. Meta-regression and sensitivity analyses were also performed to identify additional predictors of outcome and to assess the influence of trial design.

**Results:**

Five trials (3,841 patients) were gathered. A significant interaction according to treatment line was found for progression-free survival (PFS, p = 0.027); PFS was significantly improved for 1^st ^line (Hazard Ratio, HR 0.68, p < 0.0001), with a 1-yr absolute difference (AD) of 8.4% (number needed to treat, NNT 12). A non-significant trend was found in overall survival (OS), and in PFS for 2^nd ^line. Responses were improved with the addition of bevacizumab, without interaction between 1^st ^line (Relative Risk, RR 1.46, p < 0.0001) and 2^nd ^line (RR 1.58, p = 0.05). The most important toxicity was hypertension, accounting for a significant AD of 4.5% against bevacizumab (number needed to harm, NNH 22). Other significant, although less clinically meaningful, adverse events were proteinuria, neurotoxicity, febrile neutropenia, and bleeding. At the meta-regression analysis for 1^st^-line, more than 3 metastatic sites (p = 0.032), no adjuvant chemotherapy (p = 0.00013), negative hormonal receptor status (p = 0.009), and prior anthracyclines-exposure (p = 0.019), did significantly affect PFS.

**Conclusions:**

Although with heterogeneity, the addition of bevacizumab to 1^st^-line chemotherapy significantly improves PFS, and overall activity. Hypertension should be weighted with the overall benefit on the individual basis.

## Introduction

Breast cancer is the cancer with the highest incidence in women, and the major cause of death worldwide [1,2]. About 6% of patients with breast cancer present with advanced disease *ab initio*, while 40% of patients with localized disease subsequently develop distant metastases [2].

Despite numerous advances in early diagnosis and treatment in local and systemic, metastatic breast cancer remains an incurable disease and the main objective of therapy is both the prolongation of survival and the improvement of associated symptoms (palliative intent), with particular reference to delay the onset of symptoms, improvement in progression-free survival (dominant clinical endpoint used to support marketing authorizations in this setting), and improvement of quality of life [3].

Metastatic breast cancer is a heterogeneous disease whose evolution is difficult to predict. Choosing the best treatment must necessarily be based to balance different aspects of patient characteristics, the disease characteristics and possible adjuvant treatment received (cumulative dose of anthracyclines, long-term toxic effects, possible administration of taxanes and/or trastuzumab)[4]. As a future perspective, the combination of clinical and molecular factors will guide the clinician in identifying the most effective therapy for a given patient, leaving more space and giving more importance to the molecular characteristics of cancer [5,6].

Angiogenesis represents an important step in the pathogenesis, invasion, progression and development of metastatic phenotype of breast cancer and is regulated by pro-angiogenic factors such as vascular endothelial growth factor (VEGF)[7]. High expression levels of VEGF are associated with a poor prognosis and reduced survival in patients with breast cancer [8,9]. In this context, the theoretical block of tumor neo-vascularization be realized by monoclonal antibodies to factor soluble serum VEGF to its receptor or VEGFR (in different isoforms) or small molecules directed to the tyrosine-kinase receptor that appears to be a valid rationale for setting effective therapies [10]. Bevacizumab is a humanized anti-VEGF antibody approved in combination with paclitaxel for first line treatment of advanced HER2-negative breast cancer.

Although bevacizumab showed modest benefits as single agent, numerous preclinical studies have demonstrated synergy between anti-angiogenic therapy and chemotherapy [12]. The addition of Bevacizumab to chemotherapy in patients with HER-2 negative breast cancer is now one of the most viable treatment options, as the combination studies so far presented and published show that this association is able to increase the PFS and objective response [13-16].

In order to explore the magnitude of the benefit of adding Bevacizumab to chemotherapy for metastatic breast cancer with particular attention to safety, we conducted a meta-analysis.

## Methods

The analysis was conducted following 4 steps: definition of the outcomes (definition of the question the analysis was designed to answer), definition of the trial selection criteria, definition of the search strategy, and a detailed description of the statistical methods used [17,18].

### Outcome definition

The combination of chemotherapy and Bevacizumab (Beva) was considered as the experimental arm and chemotherapy as the standard comparator. Analysis was conducted in order to find significant differences in primary and secondary outcomes. Primary outcomes for the magnitude of the benefit analysis were both the Progression Free Survival (PFS: time between randomization and progression or death from any cause) and the overall survival (OS: time between randomization and death for any cause). Secondary end-points were: overall response rate (ORR), and grade 3-4 toxicities.

### Search strategy

Deadline for trial publication and/or presentation was June 30^th^, 2010. Updates of Randomized Clinical Trials (RCTs) were gathered through Medline (PubMed: http://www.ncbi.nlm.nih.gov/PubMed), ASCO (American Society of Clinical Oncology, http://www.asco.org), ESMO (European Society for Medical Oncology, http://www.esmo.org), FECS (Federation of European Cancer Societies, http://www.fecs.be), and SABCS (San Antonio Breast Cancer Symposium, http://www.sabcs.org) website searches. Key-words used for searching were: advanced/metastatic breast cancer; chemotherapy; Bevacizumab; randomized; randomized; meta-analysis; meta-regression; pooled analysis; phase III; comprehensive review, systematic review. In addition to computer browsing, review and original papers were also scanned in the reference section to look for missing trials. Furthermore, lectures at major meetings (ASCO, ESMO, ECCO, and SABCS) having 'advanced or metastatic breast cancer' as the topic were checked. No language restrictions were applied.

### Trial identification criteria

All prospective phase III RCTs published in peer-reviewed journals or presented at the ASCO, ECCO, ESMO and ASTRO meetings until June 2010, in which patients with advanced or metastatic breast cancer were prospectively randomized to chemotherapy with or without Bevacizumab were gathered, regardless of treatment lines.

### Data extraction

Hazard Ratios (HR) for PFS and OS and the number of events for secondary end-points were extracted; the last trial's available update was considered as the original source. All data were reviewed and separately computed by four investigators (F.Cu., E.B., I.S., and D.G.).

### Data synthesis

HRs were extracted from each single trial for primary end-points [19,20], and the log of relative risk ratio (RR) was estimated for secondary endpoints [21]; 95% Confidence Intervals (CI) were derived [22]. A random-effect model according to DerSimonian-Laird method was preferred to the fixed, given the known clinical heterogeneity of trials; a Q-statistic heterogeneity test was used. Absolute benefits for each outcome were calculated (i.e. absolute benefit = exp {HR or RR × log[control survival]} - control survival [23]; modified by Parmar and Machin [24]). The number of patients needed to treat (or to harm one in case of toxicity) for one single beneficial patient was determined (NNT or NNH: 1/[(Absolute Benefit)/100]) [25]. Results were depicted in all figures as conventional meta-analysis forest plots. In order to find possible correlations between outcome effect and negative prognostic factors (selected among trials' reported factors: > 3 sites, no adjuvant CT, visceral site, hormonal receptors negative (RN), prior taxanes, T or anthracyclines, A) a meta-regression approach was adopted (i.e. regression of the selected predictor on the Log HR/RR of the corresponding outcome). Calculations were accomplished using the Comprehensive Meta-Analysis Software, version v. 2.0 (CMA, Biostat, Englewood, NJ, USA).

## Results

### Selected trials

**Figure 1:**
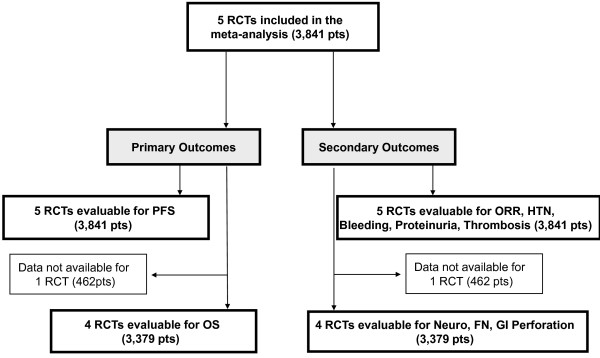
**Outline of the search - Flow diagram**. RCTs: randomized clinical trials; pts: patients; PFS: progression free survival; OS: overall survival; ORR: overall response rate; HTN: hypertension; neuro: neurotixicity; FN: febrile neutropenia; GI: gastro-intestinal.

Five trials (3,841 patients) were identified (Figure 1) [13,14,16,26,27], all included in the meta-analysis, and evaluable for PFS (primary outcome). The patients' sample for each trial ranged from 462 to 736 patients (Table 1). One trial was conducted with a double comparison [16]. Trials characteristics are listed in Table 1; 2 RCTs evaluated the addition of Bevacizumab as second line treatment [26,27], and one of these included patients who received 2 or more regimens of chemotherapy for metastatic disease [27]. One trial (462 patients) did not report survival data [27], so 4 RCTs were evaluable for OS (3,379 patients). With regard to secondary outcomes, all RCTs were evaluable for ORR, HTN, Bleeding, Proteinuria and Thrombosis; 4 RCTs (3,379 patients) were evaluable for Neurotoxicity, Febrile Neutropenia, Gastro-intestinal perforation [13,14,16,26]. With regard to the meta-regression analysis, 2 trials did not report data of two previous adjuvant chemotherapy [27], 1 trial did not refer to overall visceral disease rate [14], 1 to negative hormonal receptors [27], and 1 did not report data for previous treatment either with taxanes and anthracyclines [26].

**Table 1:** Trials' Characteristics

Authors	Pts	Prior chemotherapy lines for metastatic disease	Arms	> 3 sites	No adjuvant Chemo	Visceral site	Hormonal Receptors Negative (RN)	Prior taxanes (T)	Prior Anthra (A)
*Miller et al*	462	Mostly 1-2	Cap (2,500 mg/m^2^/day, days 1-14) Cap (2,500 mg/m^2^/day, days 1-14) + Beva (15 mg/kg)	49.7%	NR	78.7%	NR	100%	100%

*Gray et al*	722	0	wPac (90 mg/m^2 ^day 1, 8 and 15)wPac (90 mg/m^2 ^day 1, 8 and 15)+ Beva (10 mg/kg)	45.7%	34.2%	62.2%	36.7%	14.9%	37.2%

*Miles et al*	736	0	Doc (100 mg/m^2^) Doc (100 mg/m^2^)+ Beva 7.5 (7.5 mg/kg) Doc (100 mg/m^2^)+ Beva 15 (15 mg/kg)	35.0% 33.4%	54.8% 54.9%	NR	17.1% 17.1%	14.9% 16.2%	53.7% 53.5%

*Dieras et al*	622 615	0	A/T A/T + Beva (15 mg/kg) Cap (2,000 mg/m^2^/day, days 1-14) Cap (2,000 mg/m^2^/day, days 1-14) + Beva (15 mg/kg)	54.5% 27.8%	45.2% 43.9%	70.4% 68.8%	24.0% 23.6%	15.0% 39.5%	29.9% 62.9%

*Bruwski et al*	684	1	Chemo Chemo + Beva	45.3%	NR	73.1%	27.7%	NR	NR

Pt: patients; RN: receptor negative; T: taxanes (3-weekly Docetaxel or protein-bound paclitaxel); Anthra (A): anthracyclines (various regimens: AC, EC, FAC, FEC); Cap: capecitabine; Beva: Bevacizumab; NR: not reported; wPac: weekly paclitaxel; Doc: docetaxel; Chemo: various chemotherapies.

### Combined Analysis

**Figure 2:**
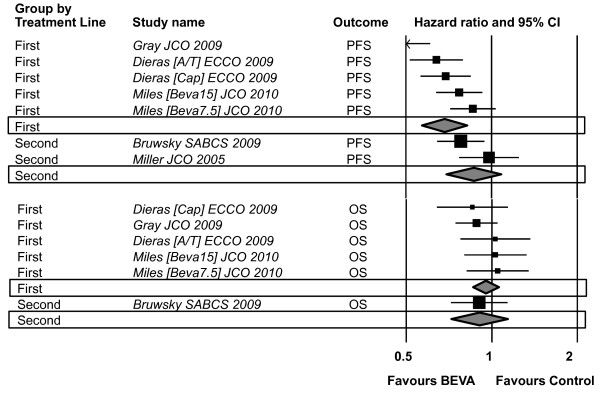
**Combined Results - Efficacy Outcomes (PFS, OS)**. CI: confidence intervals; A: anthracyclines; T: taxanes; Cap: capecitabine; Beva: bevacizumab; PFS: progression free survival; OS: overall survival.

With regard to the primary outcomes, the addition of Bevacizumab to chemotherapy increased PFS in patients untreated for advanced disease (HR 0.68, 95% CI 0.56, 0.81, p = 0.0001), with an absolute benefit of 8.4%, corresponding to 12 patients to be treated for one to benefit, although with significant heterogeneity (p = 0.0001) (Table 2) (Figure 2) . A significant interaction according to treatment lines for PFS was found (p = 0.027), given the non significant difference between the 2 arms in second line setting (HR 0.86, 95% CI 0.69, 1.07, p = 0.19). No significant differences were found in OS in favor of Bevacizumab regardless of the treatment lines (interaction test p = 0.69) (Table 2). Overall response were significantly higher in the Bevacizumab arm, regardless of treatment lines (interaction test p = 0.48), with an absolute difference of 11.5% and 8.4% for first and second line, respectively, corresponding to 8-9 and 12 patients to be treated for one to benefit (Table 2). Significant adverse events for patients receiving Bevacizumab are listed in table 3. The highest significant difference against the administration of Bevacizumab was HTN, corresponding to 22 patients to be treated for one experiencing the adverse events, although with significant heterogeneity (p = 0.0001). According to the performed meta-regression analysis, more than 3 involved sites, absence of adjuvant chemotherapy, negative hormonal receptor status and prior administration of anthracyclines are significant predictors of PFS benefit (Table 4). As shown in single trials as well [14,15], prior exposure to taxanes did not compromise the efficacy of Bevacizumab.

**Table 2:** Combined efficacy and activity results

Outcomes	Pts (RCTs)	HR/RR (95% CI)	*p-value*	Het. (*p*)	AD (%)	NNT
**PFS**						
1^st ^line	2,695 (3)	0.68 (0.56, 0.81)	*0.0001*	*0.0001*	8.4	12
2^nd ^line	1,146 (2)	0.86 (0.69, 1.07)	*0.19*	*0.14*	-	-
**OS**						
1^st ^line	2,695 (3)	0.95 (0.85, 1.05)	*0.338*	*0.64*	-	-
2^nd ^line	684 (1)	0.90 (0.71, 1.14)	*0.38*	*1.00*	-	-
**ORR**						
1^st^-line	2,695 (3)	1.46 (1.21, 1.77)	*< 0.0001*	*0.008*	11.5	8-9
2^nd^-line	1,146 (2)	1.58 (1.00, 2.52)	*0.05*	*0.092*	8.4	12

Pts: patients; RCTs: randomized clinical trials; HR: hazard ratio; RR: relative risk; CI: confidence intervals; Het.: heterogeneity; p: p-value; AD: absolute difference; NNT: number needed to treat.

**Table 3:** Significant Toxicities results

Toxicity	Pts (RCTs)	RR (95% CI)	p-value	Het. (p)	AD (%)	NNH
Hypertension	3,841 (5)	5.15 (1.60, 16.6)	*0.006*	*< 0.0001*	4.5	22

Proteinuria	3,841 (5)	9.55 (3.44, 26.5)	*< 0.0001*	*0.96*	0.4	250

Neurotoxicity	3,379 (4)	1.20 (1.01, 1.43)	*0.044*	*0.61*	2.6	39

Febrile Neutropenia	3,379 (4)	1.39 (1.07, 1.83)	*0.015*	*0.60*	2.1	46

Bleeding	3,841 (5)	3.05 (1.13, 8.23)	*0.028*	*0.56*	0.6	175

Pts: patients; RCTs: randomized clinical trials; HR: hazard ratio; CI: confidence intervals; Het.: heterogeneity; p: p-value; AD: absolute difference; NNH: number needed to harm.

**Table 4:** Meta-regression Analysis

Outcome	Predictor p-value					
	
	> 3 sites	No adjuvant Chemo	Visceral site	Hormonal Receptors Negative	Prior taxanes	Prior Anthra
PFS	***0.032***	***0.00013***	***0.03***	***0.009***	*0.96*	***0.019***

OS	*0.99*	*0.18*	*0.56*	*0.66*	*0.45*	*0.91*

Anthra (A): anthracyclines PFS: progression free survival; OS: overall survival.

## Discussion

The addition of Bevacizumab to chemotherapy is considered one of the most viable treatment options in patients with HER-2 negative metastatic breast cancer, as distinct randomized studies so far presented and published consistently showed that this association resulted in significantly improved overall response rate and PFS. Notably, the therapeutic benefit was observed in all subgroup examined. Nevertheless, the issue of adding Bevacizumab to 1^st ^line chemotherapy for advanced breast cancer is still open, given the recent concerns pointed out by the US Food and Drug administration (FDA), with specific regards to the lack of significant benefit in OS, and the toxicity profile. Moreover, the regulatory panel withheld the indication for breast cancer, and the final decision is still pending. The main question raised up by the regulatory committee refers to the eventual amount of benefit related to the addition of Bevacizumab. For this reason, a cumulative analysis specifically designed to weight that became mandatory.

The data presented herein show a statistically significant advantage in terms of either progression-free and responses, with an overall absolute benefit of 8% (Table 2). The relative risk reduction in favor of the addition of 1^st ^line Bevacizumab is 32%, and 12 patients are needed to treat in order to see one patient who significantly benefit. This amount of benefit well compares with the benefits of other important therapeutic choices such as the addition of taxanes for the 1^st ^line treatment of metastatic breast cancer, where the advantage in terms of relative risk is about 10%.

From a global perspective, the hazard ratios for PFS obtained in the current analysis compare well with those obtained in other studies that have investigated the addition of another drug in the taxane-based chemotherapy. In the study of Albain et al [28], the addition of gemcitabine to paclitaxel for advanced breast cancer after adjuvant anthracyclines based chemotherapy, the HR in terms fir the time to progression is 0.70 [28]. In the phase III trial evaluating the addition of capecitabine to docetaxel in the same setting of patients, the HR for time to disease progression is 0.65 [29].

Taking into account the different approaches to treatment such as chemotherapy combination versus single agent therapy for first line treatment of metastatic patients with breast cancer, the HR for taxanes based combinations compared with control arm was 0.92 for PFS [30]. Also with regard to the events of severe toxicities that are observed in studies that explore the benefits determined by the polychemotherapy compared to single drug therapy, are well comparable with the increase in hypertension that occurs in patients treated with bevacizumab.

With regard to the concerns regarding the interpretation of those trials providing a significant (sometimes small) benefit in intermediate end-points (such as PFS) without any advantage in late-outcomes (such as OS), a recent original work has been published, trying to weight the impact of the post-progression survival (SPP, as the difference between OS and PFS) [31]. To this purpose, simulation methods have been used to generate clinical 2-arms studies with a median PFS of 6 and 9 months, respectively. The authors indicated that OS represents a reasonable primary endpoint when the SPP is short, while when the SPP is long, that dilutes the variability of the OS, which may consequently loose the eventual statistical significance. This particular effect is especially true for those diseases where the SPP is longer than 1 year. In a context of effective treatments, such as advanced breast cancer, when a clinical trial shows a significant PFS benefit, the absence of a statistically advantage for OS does not necessarily imply the absence of a late-survival improvement [31].

Two meta-analysis analyzed the effect of the addition of Bevacizumab to chemotherapy in metastatic breast cancer [32,33] in over 3,000 patients in three randomized trials. showing a statistically significant increase in PFS, resulting in a reduced risk of progression of about 30%. In the meta-analysis conducted by Valachis et al, improved PFS was statistically significant only in the subgroup of patients receiving taxanes (or anthracyclines in a part of the study RIBBON-1) in combination with Bevacizumab [33], this advantage not seem to get in combination with capecitabine, although the latter are grouped in heterogeneous populations with regard to the treatment line. In the meta-analysis conducted by Lee et al, with populations more correctly grouped by line of treatment rather than medication, the benefit of the addition of Bevacizumab in PFS is restricted to first-line treatment [32]. Moreover, this analysis shows a marginal but statistically significant benefit in overall survival in first line.

At the last ESMO meeting, a meta-analysis of 530 elderly patients (older than 65 years) enrolled in the randomized trials ECOG 2100, AVADO and RIBBON-1, was presented [34]. Although that represent a subgroup analysis, even in these featured advanced breast cancer patients' sample, bevacizumab in combination with chemotherapy was associated with significantly improved PFS versus chemotherapy alone (HR 0.67, p = 0.0030). Hypertension was more frequent with the addition of bevacizumab, as expected; besides, no differences according to age were found.

Another relevant issue that emerges from our analysis is that the prior exposure to treatments containing taxanes does not affect the efficacy of bevacizumab (Table 4). Indeed, the meta-regression analysis for either PFS or OS clearly indicates that no significant correlation exists between the efficacy of bevacizumab and taxanes pre-treatment (p = 0.96 and p = 0.45, respectively). This finding is consistent with the ECOG-2100 and AVADO previous release [14,15], and with the recently presented meta-analysis of patients from studies ECOG-2100, AVADO and RIBBON-1, previously treated with taxanes (paclitaxel, docetaxel or paclitaxel protein-bound) [35]. This analysis included only 311 patients from the group of patients treated with taxanes of the RIBBON-1 and AVADO who received bevacizumab 15 mg/kg. The addition of bevacizumab led to an improvement in PFS from 6.2 to 10.6 months (HR 0.50, 95% CI 0.36-0.69). In line with the data of the single trials and our analysis, the authors conclude that patients pretreated with taxanes are good candidates for retreatment with bevacizumab and taxane [35].

With regard to serious adverse events, the main significant toxicity against the addition of bevacizumab was hypertension (Table 3); this represents a common finding in all disease setting when this monoclonal antibody is adopted. Our analysis shows that a weighted average of 4.5% difference between the control arm and patients undergoing bevacizumab was found, corresponding to 22 patients to be treated for one harmed (Table 3). These data are in line with those recently reported in two further cumulative analyses on the individual patients' basis, where hypertension seems to occur with different rates according to the chemotherapeutic bevacizumab is combined with [34,35]. Indeed, the initial 14-17% rate reported in the ECOG-2100 trial should be carefully evaluated, given the adoption of paclitaxel on a weekly basis (with its steroid pre-medication) could have biased the specific toxicity rate. The other significant toxicities seem to occur rarely, and in particular those toxicities supposed to be bevacizumab-related (i.e. proteinuria, bleeding) require 175-250 patients to be treated for one to be harmed. From a very practical perspective, in order to weight the relative severities of positive and negative events, breast cancer patients receiving bevacizumab in addition to chemotherapy have 'likelihood to be helped and harmed' (LHH) of 2-20 [36]; that means that patients receiving bevacizumab are from 2 to 20 times more likely to be helped than armed.

Recently, other anti-angiogenesis drugs have been studied in randomized trials for locally advanced or metastatic breast cancer [37-39]. In the SOLTI-0701 study, patients randomized to the combination of sorafenib and capecitabine showed a median PFS of 6.4 months, compared to the 4.1 months achieved by the patients who received capecitabine alone (HR 0.58, p = 0.0006) [38], although with a higher incidence of serious adverse events (hand-foot syndrome 45% versus 13%). A further randomized phase II study evaluated the efficacy and toxicity of sorafenib in addition to paclitaxel compared to paclitaxel plus placebo in patients untreated for metastatic disease, demonstrating a statistically significant improvement in PFS, TTP and responses [39]. Also for the first line treatment, the first analysis of a 3-arm randomized trial comparing paclitaxel plus placebo or bevacizumab or motesanib (small molecule inhibitor of VEGF tyrosine kinase) has been recently presented, with a median follow up of 10 months [40]. No significant differences in the primary objective of the study (the response rate), were found between the three arms, at the expense of a higher grade 3 and 4 incidence of neutropenia, hepato-biliary and gastrointestinal toxicity for patients receiving motesanib. For the second line setting of HER-2 negative patients, a recent trial randomizing patients between capecitabine and sunitinib, did not show any PFS superiority of the tyrosine kinase over capecitabine [37].

More concerning data with regard to the overall safety profile of bevacizumab have been recently released [41,42]: in the context of a literature based meta-analysis evaluating the addition of bevacizumab to chemotherapy or biologics accruing data of more than 10,000 patients regardless of the cancer type, the rate of treatment-related mortality was significantly higher in the experimental arm [41,43]. Deaths seem to be associated with hemorrhage, neutropenia and gastrointestinal perforation, with a significant interaction according to the chemotherapeutics combined (against the use of platinum or taxanes). With specific regard to breast cancer, a further meta-analysis recently showed a statistically significant higher risk of heart failure with bevacizumab [41]; both meta-analyses report no interaction according to the bevacizumab dose as a common finding. Although all these data require an individual patient data analysis for the competitive death risk evaluation, in order to clearly correlate the adverse events together, and even taking into account the heterogeneity across all studies and settings, many concerns still remain for the wide adoption of this agents [43,44].

## Conclusions

Our data in context with the other exploring the safety-efficacy balance of the addition of bevacizumab to chemotherapy for advanced breast cancer do strengthen the need of a deep analysis of the correlation between adverse events and deaths on one side, and the maximization of the efficacy by restricting the drug to those patients who will really benefit. The latest approach is far to be understood, although positive hints with regard to polymorphisms analyses are encouraging. Bevacizumab, from a clinical practice standpoint, slightly increases the efficacy of chemotherapy in HER-2 negative advanced breast cancer, although a close follow-up monitoring for adverse events must be adopted.

## Competing interests

The authors declare that they have no competing interests.

## Authors' contributions

FCu, EB, VV, PC, MM and SG conceived the analysis, and supervised the calculations; FCu, EB, IS, and DG performed the calculations in a blinded fashion; VV, FB, AF, PC, MM, CN, MR, PP, and GF participated in the trials recruitment and selection process; FCu, EB, VV, FP, AF and MM drafted and revised the manuscript; EB, PC, MM, MA, DG and FC did coordinate the overall study process and did provide the funding. All authors read and approved the final manuscript.
